# The Hearts of Man

**Published:** 1919-12

**Authors:** 


**The Hearts of Man.** By R. M. Wilson, M.B. Pp. xx., 182. Oxford Medical Publications. 1918. 6s. net.—The writer has made observations on the normal living subject, and sets out to prove a physiological theory. He commences by demonstrating that in "starting" from surprise, or at the beginning of all effort, the abdominal muscles contract in such a way as to drive as much blood as possible derived from the liver and ductless glands through the muscles and brain. Not only the heart, but also the pulmonary, abdominal and other vascular systems have a propulsive power of their own, and these "peripheral sphincters" beat synchronously with the heart. Thus there are five "hearts"—the right auricle-ventricle, the left auricle-ventricle, and the arteriole-capillary systems of the lungs, abdominal viscera and skin. The five "hearts" are joined up by larger vessels, and in these vessels peristalsis occurs, as evidenced by the anacrotic and dicrotic waves of the pulse tracing. The "hearts" are activated by adrenalin, and inhibited by the vagus or "dilator sympathetic"; the great vessels are inhibited by adrenalin, and activated by the vagus or "dilator sympathetic." The theory is adversely criticised in part by Sir James Mackenzie and Professor Bayliss, and it can scarcely be accepted as a whole. For instance, rabbit's aorta is constricted by adrenalin, and is often used as an adrenalinoscope in virtue of this fact; it is not inhibited as suggested in the book before us. The study is, however, worth consideration by those interested in the physics of the circulation.

